# One-Step Robust Low-Rank Subspace Segmentation for Tumor Sample Clustering

**DOI:** 10.1155/2021/9990297

**Published:** 2021-12-08

**Authors:** Jian Liu, Yuhu Cheng, Xuesong Wang, Shuguang Ge

**Affiliations:** School of Information and Control Engineering, China University of Mining and Technology, Xuzhou 221116, China

## Abstract

Clustering of tumor samples can help identify cancer types and discover new cancer subtypes, which is essential for effective cancer treatment. Although many traditional clustering methods have been proposed for tumor sample clustering, advanced algorithms with better performance are still needed. Low-rank subspace clustering is a popular algorithm in recent years. In this paper, we propose a novel one-step robust low-rank subspace segmentation method (ORLRS) for clustering the tumor sample. For a gene expression data set, we seek its lowest rank representation matrix and the noise matrix. By imposing the discrete constraint on the low-rank matrix, without performing spectral clustering, ORLRS learns the cluster indicators of subspaces directly, i.e., performing the clustering task in one step. To improve the robustness of the method, capped norm is adopted to remove the extreme data outliers in the noise matrix. Furthermore, we conduct an efficient solution to solve the problem of ORLRS. Experiments on several tumor gene expression data demonstrate the effectiveness of ORLRS.

## 1. Introduction

Tumor is a group of cells that have undergone unregulated growth and often form a mass or lump. It is critical to reveal the pathogenesis of cancer by analyzing tumor gene expression data. The advances of various sequencing technologies have made it possible to measure the expression levels of thousands of genes simultaneously [1]. Increasingly, one challenge is how to interpret these gene expression data to gain insights into mechanisms of tumors [2]. Many advanced machine learning algorithms [3–9] have thus been proposed to analyze various data. Among them, clustering can be used for discovering tumor samples with similar molecular expression patterns [10, 11].

Many traditional clustering methods, such as hierarchical clustering (HC) [12, 13], self-organizing maps (SOM) [14], nonnegative matrix factorization (NMF) [15, 16], and principal component analysis (PCA) [17–20] have been used for gene expression data clustering. The gene expression data often contains structures that can be represented and processed by some parametric models. The linear subspaces are possible to characterize a given set of data since they are easy to calculate and often effective in real applications. The subspace methods, such as NMF, are essentially based on the assumption that the data is approximately drawn from a low-dimensional subspace. In recent years, these methods have been gaining much attention. For example, Yu et al. proposed a correntropy-based hypergraph regularized NMF (CHNMF) method for clustering and feature selection [21]. Specifically, the correntropy is used in the loss term of CHNMF instead of the Euclidean norm to improve the robustness of the algorithm. And, CHNMF also uses the hypergraph regularization to explore the high-order geometric information in more sample points. Jiao et al. proposed a hypergraph regularized constrained nonnegative matrix factorization (HCNMF) method for selecting differentially expressed genes and tumor sample classification [22]. HCNMF incorporates a hypergraph regularization constraint to consider the higher order data sample relationships. A nonnegative matrix factorization framework based on multisubspace cell similarity learning for unsupervised scRNA-seq data analysis (MscNMF) was proposed by Wang et al. [23]. MscNMF can learn the gene features and cell features of different subspaces, and the correlation and heterogeneity between cells will be more prominent in multisubspaces, resulting in the final cell similarity learning will be more satisfactory.

However, real data rarely can be well represented by a single subspace. A more reasonable model is to assume that the data are lying near multiple subspaces (i.e., the data are considered as samples approximately drawn from a mixture of multiple low-dimensional subspaces). Subspace clustering (or segmentation) has been proposed to improve clustering accuracy. It is assumed that the data points are drawn from the combination of multiple low-dimensional subspaces. The goal of subspace clustering is to obtain such multiple low-dimensional subspaces with each subspace corresponding to a cluster. Subspace clustering has obtained promising results in previous studies, and subspace clustering methods have been found widespread applications in many areas, such as pattern recognition [24], image processing [25], and bioinformatics [26].

When the data are clean, i.e., the samples can be strictly drawn from multiple subspaces, several existing methods, such as sparse subspace clustering (SSC) [27], low-rank representation (LRR) [5], and low-rank model with discrete group structure constraint (LRS) [28], are able to solve the subspace clustering problem. SSC clusters the data drawn from multiple low-dimensional subspaces based on sparse representation (SR) [29]. Since low-rank structure can well perform matrix recover, the multiple subspaces can be exactly recovered by LRR. Recently, many excellent works based on low-rank representation are published. For example, Tang et al. proposed a multiview subspace clustering model by learning a joint affinity graph for multiview subspace clustering based on low-rank representation with diversity regularization and rank constraint [30]. This method can effectively suppress redundancy and enhance the diversity of different feature views. In addition, the cluster number is used to promote affinity graph learning by using a rank constraint. In [31], an unsupervised linear feature selective projection (FSP) method was proposed for feature extraction with low-rank embedding and dual Laplacian regularization. FSP can take advantage of the inherent relationship between data and can effectively suppress the influence of noise. LRR have two steps in the clustering task: building the affinity matrix and performing spectral clustering. How to define an excellent affinity matrix is crucial. Furthermore, the clustering problem will be transformed into a segmentation problem of graph by using spectral clustering. The choice of segmentation criteria will directly affect the clustering results. To address the above concerns, LRS directly grasps the indicators of different subspaces via the discrete constraint. As a result, multiple low-rank subspaces can be obtained clearly. Furthermore, Nie et al. introduced a piecewise function to relax the rank constraint which makes LRS better at handling the noisy dataset than the preliminary version [32].

As pointed out in [33], one major challenge of subspace clustering is to deal with the outliers that exist in data. Therefore, robust subspace clustering has become an active research topic. To address the robustness issue, the main idea is to explore the *L*_2,1_-norm based objective functions since the nonsquared residuals of *L*_2,1_-norm can reduce the effects of data outliers. In [34, 35], the *L*_2,1_-norm is adopted in robust PCA (RPCA) for detecting outliers. In [33], Liu et al. proposed a robust LRR model via *L*_2,1_-norm for subspace clustering. Although the *L*_2,1_-norm is robust to outliers, it still suffers from the extreme data outliers. The *L*_2,1_-norm just reduces, not completely removes, the effects of the outliers. Capped norm is a more robust strategy than *L*_2,1_-norm due to the fact that it can remove the effects of the outliers. It has been recently studied in many applications [36, 37].

In this paper, a one-step robust low-rank subspace segmentation (ORLRS) method via the discrete constraint and capped norm is proposed for clustering tumor sample. For a data set **X** ∈ *ℝ*^*m*×*n*^ with *m* genes and *n* samples, a low-rank representation matrix **A** ∈ *ℝ*^*m*×*n*^ and a noise matrix **E** ∈ *ℝ*^*m*×*n*^, i.e., **X**=**A**+**E**, are being sought. The low-rank representation of the *i*-th subspace **A**_*i*_ can be denoted as rank(**A**_*i*_). Here, we impose the discrete constraint on a diagonal matrix **I**_*i*_ ∈ *ℝ*^*n*×*n*^ to obtain the low-rank representation rank(**XI**_*i*_), where **I**_*i*_⊆{0,1} and ∑_*i*=1_^*c*^**I**_*i*_=**I** (*c* is the number of total subspaces and **I** is an identify matrix). The indicators of the *i*-th cluster are included in **I**_*i*_. In contrast to traditional low-rank based models, we can directly learn the cluster indicators. To avoid trivial solutions and approximate the low-rank constraint, the rank of all subspace simultaneously can be minimized as ∑_*i*=1_^*c*^(‖**AI**_*i*_‖_*S*_*p*__^*k*^)^2^, where ‖•‖_*S*_*p*__^*k*^ denotes the Schatten *p*-norm which has a better relaxation than the nuclear norm [38]. For the noise matrix **E**, capped norm is used to improve the robustness. We define *θ* as a thresholding parameter for choosing the extreme data outliers, and then the capped norm of **E** can be formulated as ‖**E**‖_Capped_=min_**E**_∑_*i*=1_^*n*^min{‖**E**_*i*_‖_2_, *θ*}. This function treats **E**_*i*_ equally if ‖**E**_*i*_‖_2_ is smaller than *θ*. Hence, it is more robust to outliers than *L*_2,1_-norm. Meanwhile, we derive an efficient optimization algorithm to solve ORLRS with a rigorous theoretical analysis.

The main contributions of our paper are given as follows:

  ① Compared with traditional low-rank representation-based methods, ORLRS can obtain the clustering result directly by learning a subspace indicator matrix from the low-rank representation matrix without spectral clustering. This avoids the graph construction process in spectral clustering and makes the clustering process simpler.
  ② We introduced the capped norm into our model and formed a novel objective function for the gene expression data clustering task. Capped norm is used to constrain the noise matrix to improve the robustness of ORLRS.
  ③ Optimizing the objective function of ORLRS is a nontrivial problem, thus we derive a new optimization algorithm to solve the problem. Furthermore, we have also given a rigorous convergence analysis of ORLRS.

The remainder of the paper is structured as follows. In Section 2, the proposed ORLRS is presented, and the theoretical analysis of the proposed method is provided. Experimental results are presented in Section 3. In Section 4, the conclusions are given.

## 2. Methods

We start with a brief introduction of several classical clustering methods. Then, the proposed ORLRS is presented, and the optimal solution and convergence analysis of ORLRS is provided.

### 2.1. Subspace Clustering via LRR

Denote **X** ∈ *ℝ*^*m*×*n*^ as a data set with *m* features and *n* samples. LRR can be defined as

(1) $$\begin{array}{c} \underset{A,E}{\min}{\left\|A\right\|}_{*}+\lambda {\left\|E\right\|}_{2,1},\\ \text{s.t.}X=DA+E, \end{array}$$

where ‖**A**‖_*∗*_=∑_*i*_*σ*_*i*_(**A**), i.e., the nuclear norm of **A** [33], ${\left\|E\right\|}_{2,1}=\sum_{j=1}^{n}\sqrt{\sum_{i=1}^{m}E_{ij}^{2}}$ can detect outliers with column-wise sparsity, **D** is a dictionary, and *λ* > 0 is a balance parameter.

A brief explanation of LRR subspace clustering process is provided as follows. Firstly, the low-rank problem is solved by equation (1). Then, the optimal solution **A**^*∗*^ to equation (1) is used to calculate the affinity matrix by (|**A**^*∗*^|+|(**A**^*∗*^)^*T*^|)/2, where |**A**^*∗*^| is the absolute value function. Finally, the data are clustered by using spectral clustering [39].

### 2.2. One-Step Robust Low-Rank Subspace Clustering

In this paper, we propose the one-step robust low-rank subspace clustering (ORLRS) method via discrete constraint and capped norm. Different from LRR, ORLRS was proposed for clustering the data by learning the indicators.

Suppose the data matrix **X** has *c* subspaces {**X**_1_, **X**_2_,…, **X**_*c*_}, the low-rank representation of each subspace needs to be optimized. In the clustering task, we want each subspace to belong to its own cluster. To obtain a low-rank representation of each subspace, the following formula should be computed: rank(**X**_*i*_), which has trivial solution. Therefore, we need to solve the problem in another way. We define a cluster indicator matrix as **C** ∈ *ℝ*^*c*×*n*^: **C**(*i*, *j*)=1 if the *j*-th sample belongs to the *i*-th subspace, and **C**(*i*, *j*)=0 otherwise. And, the *c* diagonal matrices are defined as **I**_1_, **I**_2_,…, **I**_*c*_ ∈ **I**, where the diagonal elements of **I**_*i*_ (1 ≤ *i* ≤ *c*) are formed by the *i*-th row of **C** and **I** is the identity matrix. Then, **XI**_*i*_ can be represented as the *i*-th subspace of **X**. That is, rank(**X**_*i*_) can be rewritten as rank(**XI**_*i*_). We can get the clustering label in one step by directly optimizing **I**_*i*_ [28].

Finally, the problem of the one-step low-rank subspace clustering method can be defined as

(2) $$\begin{array}{c} \underset{{I_{i}|}_{I=1}^{c}}{\min}\sum_{i=1}^{c}{\left({\left\|XI_{i}\right\|}_{S_{p}}^{p}\right)}^{2},\\ \text{s.t.}{I_{i}|}_{I=1}^{c}\subseteq {\left\{0,1\right\}}^{n\times n},\sum_{i=1}^{c}I_{i}=I. \end{array}$$

where ‖**XI**_*i*_‖_*S*_*p*__^*p*^ is the Schatten *p*-norm of **XI**_*i*_. The clustering indicators of each subspace can be obtained from the optimized diagonal matrix **I**_*i*_*|*_*i*=1_^*c*^ directly.

However, equation (2) is sensitive to data outliers in practical problems since it does not consider the noise in data. To address the robustness problem, we represent the gene expression data **X** ∈ *ℝ*^*m*×*n*^ with *m* genes and *n* samples as the addition of low-rank representation matrix **A** ∈ *ℝ*^*m*×*n*^ and the noise matrix **E** ∈ *ℝ*^*m*×*n*^, i.e., **X**=**A**+**E**, which is the same strategy as in RPCA. Our one-step low-rank subspace clustering problem can be written as

(3) $$\begin{array}{c} \underset{A,E,{I_{i}|}_{i=1}^{c}}{\min}\sum_{i=1}^{c}{\left({\left\|AI_{i}\right\|}_{S_{p}}^{k}\right)}^{2}\;+\lambda {\left\|E\right\|}_{L},\\ \text{s.t.}X=A+E,{I_{i}|}_{i=1}^{c}\subseteq {\left\{0,1\right\}}^{n\times n},\sum_{i=1}^{c}I_{i}=I, \end{array}$$

where *λ* > 0 is a balance parameter and ‖•‖_*L*_ indicates certain regularization strategy. Note that Schatten *p*-norm is used to approximate the low-rank problem in equation (3) since it is a better relaxation for the rank constraint problem than nuclear norm [38]. The Schatten *p*-norm (0 < *p* < *∞*) of a matrix **G** ∈ *ℝ*^*m*×*n*^ was defined as ‖**G**‖_*S*_*p*__=(∑_*i*=1_^min(*m*, *n*)^*σ*_*i*_^*p*^)^1/*p*^, where *σ*_*i*_ is the *i*-th singular value of **G**. In [38], the convergence of Schatten *p*-norm with 0 < *p* ≤ 2 is proved. Here, we set 0 < 2*k* ≤ 2 to guarantee the convergence of first term in equation (3). So, the range of *k* is 0 < *k* ≤ 1.

To seek a better robustness strategy for the outliers, we adopt capped norm to regularize the noise matrix **E**, i.e., ‖**E**‖_Capped_. Then, equation (3) becomes

(4) $$\begin{array}{c} \underset{A,E,{I_{i}|}_{i=1}^{c}}{\min}\sum_{i=1}^{c}{\left({\left\|AI_{i}\right\|}_{S_{p}}^{k}\right)}^{2}+\lambda \sum_{i=1}^{n}\min\left\{{\left\|E_{i}\right\|}_{2},\theta \right\},\\ \text{s.t.}X=A+E,{I_{i}|}_{i=1}^{c}\subseteq {\left\{0,1\right\}}^{n\times n},\sum_{i=1}^{c}I_{i}=I, \end{array}$$

where *θ* > 0 is a thresholding parameter for choosing the data outliers. If the data point ‖**E**_*i*_‖_2_ > *θ*, we consider **E**_*i*_ as extreme outlier, and it is capped as *θ*. In this way, the influence of extreme outliers is fixed. For other data point ‖**E**_*i*_‖_2_ ≤ *θ*, equation (4) will minimize ∑_*i*=1_^*n*^‖**E**_*i*_‖_2_, i.e., the *L*_2,1_-norm. That is, if *θ* is set as *∞*, ‖**E**‖_Capped_ is equivalent to ‖**E**‖_2,1_. Thus, the capped norm is a more robust strategy than *L*_2,1_-norm.

As a result, ORLRS provides a more robust low-rank subspace clustering model by using capped norm. And, the clustering indicators of each subspace can be obtained from the optimized diagonal matrix **I**_*i*_*|*_*i*=1_^*c*^ directly. We will propose an efficient optimization algorithm to solve equation (4) in Section 2.3.

### 2.3. Optimization Algorithm

The objective function equation (4) of the ORLRS is nonconvex, thus jointly optimizing **A**, **E**, and **I**_*i*_*|*_*i*=1_^*c*^ is extremely difficult. The augmented Lagrange multiplier (ALM) algorithm is used to optimize equation (4). The Lagrangian function of equation (4) can be written as

(5) $$\begin{array}{c} \underset{A,E,{I_{i}|}_{i=1}^{c}}{\min}\sum_{i=1}^{c}{\left({\left\|AI_{i}\right\|}_{S_{p}}^{k}\right)}^{2}+\lambda \sum_{i=1}^{n}\min\left\{{\left\|E_{i}\right\|}_{2},\theta \right\}+\langle Y,A+E-X\rangle +\frac{\mu }{2}{\left\|A+E-X\right\|}_{F}^{2}+g\left(\Lambda ,{I_{i}|}_{i=1}^{c}\right), \end{array}$$

where **Y** is a Lagrange multiplier, *μ* > 0 is a penalty parameter, ‖•‖_*F*_ is the Frobenius norm, and *g*(Λ, **I**_*i*_*|*_*i*=1_^*c*^) encodes the constraints of **I**_*i*_*|*_*i*=1_^*c*^. We rewrite equation (5) as follows:

(6) $$\begin{array}{c} \underset{A,E,{I_{i}|}_{i=1}^{c}}{\min}\sum_{i=1}^{c}{\left({\left\|AI_{i}\right\|}_{S_{p}}^{k}\right)}^{2}+\lambda \sum_{i=1}^{n}\min\left\{{\left\|E_{i}\right\|}_{2},\theta \right\}+\frac{\mu }{2}{\left\|A+E-X+\frac{Y}{\mu }\right\|}_{F}^{2}+g\left(\Lambda ,{I_{i}|}_{i=1}^{c}\right). \end{array}$$

We divide equation (6) into three subproblems: optimizing **A** while fixing **E** and **I**_*i*_*|*_*i*=1_^*c*^, optimizing **E** while fixing **A** and **I**_*i*_*|*_*i*=1_^*c*^, and optimizing **I**_*i*_*|*_*i*=1_^*c*^ while fixing **A** and **E**.

#### 2.3.1. Fixing **E** and **I**_*i*_*|*_*i*=1_^*c*^ to Optimize **A**

Equation (6) can be simplified to

(7) $$\begin{array}{c} \underset{A}{\min}\sum_{i=1}^{c}{\left({\left\|AI_{i}\right\|}_{S_{p}}^{k}\right)}^{2}+\frac{\mu }{2}{\left\|A-B\right\|}_{F}^{2}, \end{array}$$

where **B**=**X** − **E**+(**Y**/*μ*).

**Lemma 1 .**

(Araki-Lieb-Thirring [40, 41]). For any positive semidefinite matrices **P**, **Q** ∈ *ℝ*^*n*×*n*^, *q* > 0, the following inequality holds when 0 ≤ *h* < 1:

(8) $$\begin{array}{c} \text{Tr}{\left(P^{h}Q^{h}P^{h}\right)}^{q}\leq \text{Tr}{\left(PQP\right)}^{hq}. \end{array}$$

While for *h* ≥ 1, the inequality is reversed.

Following 0 < *k* ≤ 1, ‖**G**‖_*S*_*p*__^*p*^=Tr((**G**^*T*^**G**)^*p*/2^) [28] and Lemma 1, the first term in equation (7) can be denoted as ∑_*i*=1_^*c*^Tr(**I**_*i*_(**A**^*T*^**A**)^*k*^)=∑_*i*=1_^*c*^(‖**AI**_*i*_‖_*S*_*p*__^*k*^)^2^ since **I**_*i*_*|*_*i*=1_^*c*^⊆{0,1}^*n*×*n*^. According to ∑_*i*=1_^*c*^**I**_*i*_=**I**, we convert the first term in equation (7) to

(9) $$\begin{array}{c} \sum_{i=1}^{c}\text{Tr}\left(I_{i}{\left(A^{T}A\right)}^{k}\right)=\text{Tr}\left(I_{1}{\left(A^{T}A\right)}^{k}+I_{2}{\left(A^{T}A\right)}^{k}+\cdots +I_{c}{\left(A^{T}A\right)}^{k}\right)=\text{Tr}{\left(A^{T}A\right)}^{k}. \end{array}$$

Then, equation (7) can be represented as

(10) $$\begin{array}{c} J_{1}=\underset{A}{\min}\text{Tr}{\left(A^{T}A\right)}^{k}\;+\frac{\mu }{2}{\left\|A-B\right\|}_{F}^{2}. \end{array}$$

Taking derivative w.r.t **A** and setting to zero, the above formula becomes

(11) $$\begin{array}{c} \frac{\partial J_{1}}{\partial A}=2AH+\mu \left(A-B\right)=0, \end{array}$$

where **H**=*k*(**A**^*T*^**A**)^*k*−1^. So, we can achieve the optimal **A**:

(12) $$\begin{array}{c} A=\mu B{\left(2H+\mu I\right)}^{-1}. \end{array}$$

#### 2.3.2. Fixing **A** and **I**_*i*_*|*_*i*=1_^*c*^ to Optimize **E**

Here, we can denote equation (6) as

(13) $$\begin{array}{c} \underset{E}{\min}\lambda \sum_{i=1}^{n}\min\left\{{\left\|E_{i}\right\|}_{2},\theta \right\}+\frac{\mu }{2}{\left\|E-F\right\|}_{F}^{2}, \end{array}$$

where **F**=**X** − **A**+(**Y**/*μ*). It can be easily verified that the derivative of equation (13) is equivalent to the derivative of

(14) $$\begin{array}{c} \underset{E}{\min}\lambda \sum_{i=1}^{n}o_{i}{\left\|E_{i}\right\|}_{2}^{2}+\frac{\mu }{2}{\left\|E-F\right\|}_{F}^{2}, \end{array}$$

where

(15) $$\begin{array}{c} o_{i}=\left\{\begin{array}{cc} \frac{1}{2{\left\|E_{i}\right\|}_{2}}, & \text{if }{\left\|E_{i}\right\|}_{2}<\theta ;\\ 0, & \text{otherwise}. \end{array}\right. \end{array}$$

Equation (14) can be formulated as

(16) $$\begin{array}{c} J_{2}=\underset{E}{\min}\lambda \text{Tr}\left(OE^{T}E\right)+\frac{\mu }{2}{\left\|E-F\right\|}_{F}^{2}, \end{array}$$

where **O** is a diagonal matrix with **O**_*ii*_=*o*_*i*_. The problem of equation (16) can be optimized by using the iterative reweighted optimization strategy.

When fixing **O**, taking derivative w.r.t **E** and setting it to zero, the above formulation can be written as

(17) $$\begin{array}{c} \frac{\partial J_{2}}{\partial E}=2\lambda EO+\mu \left(E-F\right)=0. \end{array}$$

So, we can obtain the optimal **E**:

(18) $$\begin{array}{c} E=\mu F{\left(2\lambda O+\mu I\right)}^{-1}. \end{array}$$

When fixing **E**, the updating rule for **O** is as follows:

(19) $$\begin{array}{c} O_{ii}=\left\{\begin{array}{cc} \frac{1}{2{\left\|E_{i}\right\|}_{2}}, & \text{if }{\left\|E_{i}\right\|}_{2}<\theta ;\\ 0, & \text{otherwise}. \end{array}\right. \end{array}$$

#### 2.3.3. Fixing **A** and **E** to Optimize **I**_*i*_*|*_*i*=1_^*c*^

We can rewrite equation (6) as

(20) $$\begin{array}{c} J_{3}=\underset{{I_{i}|}_{i=1}^{c}}{\min}\sum_{i=1}^{c}{\left({\left\|AI_{i}\right\|}_{S_{p}}^{k}\right)}^{2}+g\left(\Lambda ,{I_{i}|}_{i=1}^{c}\right). \end{array}$$

Taking derivative w.r.t **I**_*i*_*|*_*i*=1_^*c*^ and setting to zero, the above formulation can be written as

(21) $$\begin{array}{c} \frac{\partial J_{3}}{\partial {I_{i}|}_{i=1}^{c}}=\sum_{i=1}^{c}2A^{T}L_{i}AI_{i}+\frac{\partial g\left(\Lambda ,{I_{i}|}_{i=1}^{c}\right)}{\partial {I_{i}|}_{i=1}^{c}}=0, \end{array}$$

where **L**_*i*_=*k*‖**AI**_*i*_‖_*S*_*p*__^*k*^(**AI**_*i*_^2^**A**^*T*^)^(*k* − 2)/2^.

Since **L**_*i*_ depends on **I**_*i*_, an iteration-based algorithm is used to obtain the solution of equation (21). Firstly, we calculate **L**_*i*_ by using the current solution of **I**_*i*_. If **L**_*i*_ is given, the solution of **I**_*i*_ to the following objective function will satisfy equation (21):

(22) $$\begin{array}{c} \underset{{I_{i}|}_{i=1}^{c}}{\min}\;\sum_{i=1}^{c}\text{Tr}\left(I_{i}^{T}A^{T}L_{i}AI_{i}\right)\text{,}\\ \text{s.t.}\;{I_{i}|}_{i=1}^{c}\subseteq {\left\{0,1\right\}}^{n\times n},\sum_{i=1}^{c}I_{i}=I. \end{array}$$

The current solution of **I**_*i*_ can be updated according to the optimal solution to equation (22).

Denote that **Z**_*i*_=**A**^*T*^**L**_*i*_**A**, equation (22) can be written as

(23) $$\begin{array}{c} \underset{{I_{i}|}_{i=1}^{c}}{\min}\sum_{i=1}^{c}\text{Tr}\left(Z_{i}I_{i}\right)\text{,}\\ \text{s.t.}{I_{i}|}_{i=1}^{c}\subseteq {\left\{0,1\right\}}^{n\times n},\sum_{i=1}^{c}I_{i}=I. \end{array}$$

Due to **I**_*i*_*|*_*i*=1_^*c*^ are *n* × *n* diagonal matrices, the above formulation becomes

(24) $$\begin{array}{c} \underset{r_{ci}\in \left\{0,1\right\},\sum_{i=1}^{c}r_{ci}=1}{\min}\sum_{i=1}^{c}\sum_{i=c}^{n}\left(z_{ci}r_{ci}\right), \end{array}$$

where *r*_*ci*_ is the *c*-th diagonal element of matrix **I**_*i*_ and *z*_*ci*_ is the *c*-th diagonal element of matrix **Z**_*i*_. We can optimize equation (24) by

(25) $$\begin{array}{c} r_{ci}=\left\{\begin{array}{cc} 1, & \text{if }i=\arg\underset{l}{\min}\left(z_{cl}\right),\\ 0, & \text{otherwise.} \end{array}\right. \end{array}$$

The algorithm to solve the problem of ORLRS is summarized in Algorithm 1.

### 2.4. Convergence Analysis

In this section, the convergence analysis of the proposed algorithm will be proved.

**Theorem 1 .**

At each iteration, the updating rule in Algorithm 1 for matrix **A** while fixing others will monotonically decrease the objective value in equation (4) when 0 < *k* ≤ 1.

**Proof**

It can be verified that equation (12) is the solution to the following problem:

(26) $$\begin{array}{c} \underset{A}{\min}\text{Tr}\left(A^{T}AH\right).\\ \text{s.t.}X=A+E. \end{array}$$

Then, at the *t* iteration

(27) $$\begin{array}{c} A_{t+1}=\arg\underset{A}{\min}\text{Tr}\left(A^{T}AH_{t}\right). \end{array}$$

That is,

(28) $$\begin{array}{c} \left.\text{Tr}\left(A_{t+1}^{T}A_{t+1}H_{t}\right)\right)\leq \text{Tr}\left(A_{t}^{T}A_{t}H_{t}\right). \end{array}$$

Equation (28) can be converted to

(29) $$\begin{array}{c} k\text{Tr}\left(A_{t+1}^{T}A_{t+1}{\left(A_{t}^{T}A_{t}\right)}^{k-1}\right)\;\leq \left.k\text{Tr}\left(A_{t}^{T}A_{t}{\left(A_{t}^{T}A_{t}\right)}^{k-1}\right)\right)\text{ ,} \end{array}$$

according to Lemma 2 in [38].

**Lemma 2 .**

For any positive definite matrices **P**, **P**_*t*_ ∈ *ℝ*^*m*×*m*^, the following inequality holds when 0 < *p* ≤ 2.

(30) $$\begin{array}{c} \text{Tr}\left(P^{p/2}\right)-\frac{p}{2}\text{Tr}\left(\left(PP_{t}^{\left(p-2\right)/2}\right)\right.\leq \text{Tr}\left(P_{t}^{p/2}\right)-\frac{p}{2}\text{Tr}\left(\left(P_{t}P_{t}^{\left(p-2\right)/2}\right)\right., \end{array}$$

Note that, here, we set 0 < *k* ≤ 1, so equation (30) is equivalent to

(31) $$\begin{array}{c} \text{Tr}\left(P^{k}\right)-k\text{Tr}\left(\left(PP_{t}^{k-1}\right)\right.\leq \text{Tr}\left(P_{t}^{k}\right)-k\text{Tr}\left(\left(P_{t}P_{t}^{k-1}\right)\right., \end{array}$$

Then, we have

(32) $$\begin{array}{c} \text{Tr}\left({\left(A^{T}A\right)}^{k}\right)-k\text{Tr}\left(A^{T}A{\left(A_{t}^{T}A_{t}\right)}^{k-1}\right)\\ \leq \text{Tr}\left({\left(A_{t}^{T}A_{t}\right)}^{k}\right)-\left.k\text{Tr}\left(A_{t}^{T}A_{t}{\left(A_{t}^{T}A_{t}\right)}^{k-1}\right)\right). \end{array}$$

Combining equations (29) and (32), we have

(33) $$\begin{array}{c} \text{Tr}\left({\left(A^{T}A\right)}^{k}\right)\leq \text{Tr}\left({\left(A_{t}^{T}A_{t}\right)}^{k}\right). \end{array}$$

That is to say,

(34) $$\begin{array}{c} {\left({\left\|A_{t+1}\right\|}_{S_{p}}^{k}\right)}^{2}\leq {\left({\left\|A_{t}\right\|}_{S_{p}}^{k}\right)}^{2}. \end{array}$$

Thus, the updating rule for matrix **A** in Algorithm 1 will not increase the objective value of the problem in equation (10) at each iteration *t* when 0 < *k* ≤ 1.

**Theorem 2 .**

At each iteration, the updating rule in Algorithm 1 for matrix **E** while fixing others will monotonically decrease the objective value in equation (4).

**Proof**

We fist prepare the following lemma in [37].

**Lemma 3 .**

Given $s=\left\{\begin{array}{cc} \left(1/2\left|e\right|\right), & \text{if }\left|e\right|<\theta ,\\ 0, & \text{otherwise,} \end{array}\right.$, we have the following inequality:

(35) $$\begin{array}{c} \min\left(\left|\tilde{e}\right|,\theta \right)-s{\tilde{e}}^{2}\leq \min\left(\left|e\right|,\theta \right)-se^{2}. \end{array}$$

It can be verified that equation (18) is the solution to the following problem:

(36) $$\begin{array}{c} \underset{E}{\min}\;\text{Tr}\left(OE^{T}E\right),\\ \text{s.t.}\;X=A+E. \end{array}$$

Suppose the updated **E** in Algorithm 1 is $\tilde{E}$ while fixing others. Since $\tilde{E}$ is the optimal solution to equation (4), we have

(37) $$\begin{array}{c} \lambda \text{Tr}\left(O{\tilde{E}}^{T}\tilde{E}\right)\leq \lambda \text{Tr}\left(OE^{T}E\right). \end{array}$$

According to the definition of **O**_*ii*_ in equation (19) and Lemma 3, we have

(38) $$\begin{array}{c} \lambda \sum_{i=1}^{n}\min\left({\left\|{\tilde{E}}_{i}\right\|}_{2},\theta \right)-\lambda \sum_{i=1}^{n}O_{ii}{\left\|{\tilde{E}}_{i}\right\|}_{2}^{2}\\ \leq \lambda \sum_{i=1}^{n}\min\left({\left\|E_{i}\right\|}_{2},\theta \right)-\lambda \sum_{i=1}^{n}O_{ii}{\left\|E_{i}\right\|}_{2}^{2}. \end{array}$$

Summing over equations (37) and (38) at both sides, we can obtain

(39) $$\begin{array}{c} \lambda \sum_{i=1}^{n}\min\left({\left\|{\tilde{E}}_{i}\right\|}_{2},\theta \right)\leq \lambda \sum_{i=1}^{n}\min\left({\left\|E_{i}\right\|}_{2},\theta \right). \end{array}$$

Therefore, at each iteration, the updating rule in Algorithm 1 for matrix **E** while fixing others will monotonically decrease the objective value in equation (4).

**Theorem 3 .**

At each iteration, the updating rule in Algorithm 1 for **I**_*i*_*|*_*i*=1_^*c*^ while fixing others will monotonically decrease the objective value in equation (4) when *k*=1.

**Proof**

It can be easily verified that equation (25) is the solution to the following problem:

(40) $$\begin{array}{c} \underset{{I_{i}|}_{i=1}^{c}}{\min}\sum_{i=1}^{c}{\left({\left\|AI_{i}\right\|}_{S_{p}}^{k}\right)}^{2},\\ \text{s.t.}{I_{i}|}_{i=1}^{c}\subseteq {\left\{0,1\right\}}^{n\times n},\sum_{i=1}^{c}I_{i}=I. \end{array}$$

Assume the updated **I**_*i*_ in Algorithm 1 is ${\tilde{I}}_{i}$. Since ${\tilde{I}}_{i}$ is the optimal solution to the equation (22), we can have

(41) $$\begin{array}{c} \sum_{i=1}^{c}\text{Tr}\left({\tilde{I}}_{i}^{T}A^{T}L_{i}A{\tilde{I}}_{i}\right)\leq \sum_{i=1}^{c}\text{Tr}\left(I_{i}^{T}A^{T}L_{i}AI_{i}\right). \end{array}$$

According to the definition of **L**_*i*_ in Algorithm 1, equation (41) can be written as

(42) $$\begin{array}{c} \sum_{i=1}^{c}{\left\|AI_{i}\right\|}_{S_{p}}^{k}\text{Tr}\left({\left(AI_{i}^{2}A^{T}\right)}^{\left(k-2\right)/2}A^{T}{\left({\tilde{I}}_{i}^{T}\right)}^{2}A\right)\leq \sum_{i=1}^{c}{\left({\left\|AI_{i}\right\|}_{S_{p}}^{k}\right)}^{2}. \end{array}$$

According to the Cauchy–Schwarz inequality, it can be proved that, when *p*=1, we have

(43) $$\begin{array}{c} \sum_{i=1}^{c}{\left({\left\|A{\tilde{I}}_{i}\right\|}_{S_{p}}^{k}\right)}^{2}\\ \leq \sum_{i=1}^{c}\text{Tr}\left({\left(AI_{i}^{2}A^{T}\right)}^{k/2}\right)\text{Tr}\left({\left(AI_{i}^{2}A^{T}\right)}^{\left(k-2\right)/2}A^{T}{\left({\tilde{I}}_{i}^{T}\right)}^{2}A\right)\;. \end{array}$$

Thus, combining inequations (42) and (43), we can obtain

(44) $$\begin{array}{c} \sum_{i=1}^{c}{\left({\left\|A{\tilde{I}}_{i}\right\|}_{S_{p}}^{k}\right)}^{2}\leq \sum_{i=1}^{c}{\left({\left\|AI_{i}\right\|}_{S_{p}}^{k}\right)}^{2}. \end{array}$$

Equation (44) indicates that the updating rule in Algorithm 1 for **I**_*i*_*|*_*i*=1_^*c*^ while fixing others will monotonically decrease the objective value in equation (4) during the iteration until the algorithm converges when *k*=1. In practice, the algorithm is also converged when 0 < *k* < 1. If the objective function of equation (40) is changed to ∑_*i*=1_^*c*^(‖**AI**_*i*_‖_*S*_*p*__^*k*^)^*d*^,  (*d* > 2) (**L**_*i*_ in Algorithm 1 becomes (*dk*/2)(‖**AI**_*i*_‖_*S*_*p*__^*k*^)^*d*−1^(**AI**_*i*_^2^**A**^*T*^)^(*k* − 2)/2^), the convergence is also observed [28].

As a result, the objective of equation (4) is nonincreasing under the updates of **A**, **E**, and **I**_*i*_*|*_*i*=1_^*c*^ according to Theorems 1–3, respectively. Therefore, the iteratively updating Algorithm 1 converges to a local optimal.

### 2.5. Complexity Analysis

In Algorithm 1, the most complicated calculations are **L**_*i*_=*k*‖**AI**_*i*_‖_*S*_*p*__^*k*^(**AI**_*i*_^2^**A**^*T*^)^(*k* − 2)/2^ and **Z**_*i*_=**A**^*T*^**L**_*i*_**A** in Step 3. We suppose *m* > *n* in the low-rank representation matrix **A** ∈ *ℝ*^*m*×*n*^. Firstly, **L**_*i*_ needs to be computed. Denoting the SVD of **AI**_*i*_ is **U**Σ**V**^*T*^. Computing ‖**AI**_*i*_‖_*S*_*p*__^*k*^ needs SVD of **AI**_*i*_, which takes *O*(*mn*^2^). (**AI**_*i*_^2^**A**^*T*^)^(*k* − 2)/2^ can be decomposed as **U**Σ^*k*−2^**U**^*T*^ by SVD, which takes *O*(*m*^3^). So, computing **L**_*i*_ takes *O*(*m*^3^) and computing **L**_*i*_*|*_*I*=1_^*c*^ takes *O*(*m*^3^*c*), where *c* is the number of clusters. For **Z**_*i*_, we only need to compute the diagonal elements, which takes *O*(*m*^3^). And, computing **Z**_*i*_*|*_*I*=1_^*c*^ takes *O*(*m*^3^*c*). In summary, the computational complexity of Algorithm 1 is *O*(*m*^3^*ct*), where *t* is the iteration number.

## 3. Results and Discussion

We test ORLRS on six publicly available gene expression data sets, i.e., Leukemia [42], DLBCL [43], Colon cancer [44], Brain_Tumor1 [43], Brain_Tumor2 [43], and 9_Tumors [43].

Following [28, 45–47], clustering accuracy (ACC) is a widely used evaluation method for tumor clustering. Given a data point **x**_*i*_, suppose *N*_*i*_ as the target label and *T*_*i*_ as the truth label. ACC can be denoted as [45]

(45) $$\begin{array}{c} \text{ACC}=\frac{\sum_{i=1}^{n}\varphi \left[T_{i},\text{map}\left(N_{i}\right)\right]}{n}, \end{array}$$

where *φ*(*x*, *y*)=0 if *x* ≠ *y* and *φ*(*x*, *y*)=1 if *x*=*y*, map(*N*_*i*_) maps *N*_*i*_ to the equivalent label from the raw data and *n* is the number of tumor samples.

We also evaluate the clustering performance by normalized mutual information (NMI) [48]. NMI is defined as

(46) $$\begin{array}{c} \text{NMI}=\frac{M\left(S,C\right)}{\left(\left(H\left(S\right)+H\left(C\right)\right)/2\right)}, \end{array}$$

where *M*(*S*, *C*) is the mutual information function between the true class label *C* and the clustering label *S* and *H*(·) is the entropy function. The larger the NMI value is, the better the clustering result is.

### 3.1. Gene Expression Data Sets

A brief introduction of six gene expression data sets is presented, and the detailed information of these datasets is summarized in Table 1.

  Leukemia data contain 25 cases of AML and 47 cases of ALL. It is packaged into a 7129 × 72 matrix [42].
  DLBCL data consist of 5469 genes and 77 samples. These samples include 58 patients of diffuse large B-cell lymphoma (DLBCL) and 19 patients of follicular lymphomas (FL) [43].
  The colon cancer data [44] consists of a matrix that includes 2000 genes and 62 tissues. These tissues are divided into 22 normal and 40 colon tumor samples
  Brain_Tumor1 data set consists of 5920 genes in 90 patient samples. These samples contain 5 types of histological diagnoses, i.e., 60 cases of medulloblastoma, 10 cases of malignant glioma, 10 cases of atypical teratoid/rhabdoid tumors (AT/RTs), 4 cases of normal cerebellum, and 6 cases of primitive neuroectodermal tumors (PNETs).
  The Brain_Tumor2 data set contains 10367 genes in 50 samples. It contains 4 types of malignant glioma, i.e., classic glioblastomas (CG), classic anaplastic oligodendrogliomas (CAO), nonclassic glioblastomas (NCG), and nonclassic anaplastic oligodendrogliomas (NCAO) [34].
  9_Tumors data set integrates 9 tumor types to develop a genomics-based approach to the prediction of drug response. It contains 5726 genes in 60 samples. The number of samples of 9 tumor types is shown as follows: 9 samples of non-small-cell carcinoma (NSCLC), 7 samples of colon cancer, 8 samples of breast cancer, 6 samples of ovary cancer, 6 samples of leukemia, 8 samples of renal cancer, 8 samples of melanoma, 2 samples of prostate cancer, and 6 samples central nervous system cancer (CNS).

### 3.2. Comparison Algorithms

We compare LRS [28], Ext-LRR [32], RPCA [3], PLRR [47], robust LRR [33], LatLRR [49], robust NMF [50], and K-means [51] with the proposed method for tumor clustering. In these methods, LRS is the basic version of our method to implement the one-step clustering, and Ext-LRR is a simpler and more effective extension work compared with LRS; RPCA is a classic robust learning algorithm; PLRR (projection LRR) is one of the latest subspace clustering methods for tumor sample clustering; Robust LRR and LatLRR are the best state-of-art low-rank subspace segmentation algorithms; robust NMF is a classic NMF-based method and is widely used for tumor clustering. *K*-means is the most commonly used clustering method and is embedded into many methods including PLRR, robust LRR, and LatLRR to achieve better performance. Since our proposed method is a novel one-step robust low-rank subspace clustering model, we choose these methods as our comparison algorithms.

### 3.3. Parameter Setting

Since gene expression data have the characteristics of high-dimensional and small samples, we use PCA to perform dimensionality reduction. And, we use the K-means method to initialize **I**_*i*_*|*_*i*=1_^*c*^ in the proposed ORLRS. Here, three parameters, i.e., threshold parameter *θ*, balance parameter *λ*, and low-rank constraint parameter *k*, need to be determined. In the experiment, we investigated one parameter by fixing the other two parameters. Since the initialization of **I**_*i*_*|*_*i*=1_^*c*^ will bring some uncertainty, the proposed ORLRS method run 100 times, and the average of the accuracies of 100 times is reported. The choices of parameters in the following are heuristic and might not be the best for tumor clustering.

#### 3.3.1. Determination of Threshold Parameter *θ*

In the ORLRS model, data outliers are not heuristically determined based on the magnitude. They are selected during the optimization process. The data outliers may be distinct at different iterations (with the same thresholding parameter), while we iteratively optimize the objective function of ORLRS method. When the algorithm converges, likely correct extreme data outliers can be found. So, we just need to determine one value of *θ* for each data set.

**Figure 1:**
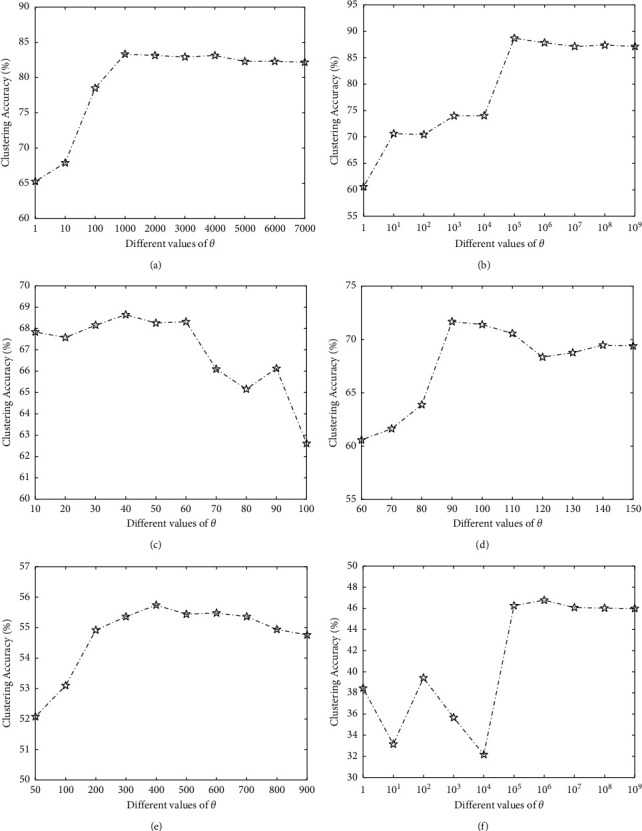
Clustering accuracies of the proposed ORLRS on six data sets with different values of *θ*. (a) Leukemia. (b) DLBCL. (c) Colon. (d) Brain_Tumor1. (e) Brain_Tumor2. (f) 9_Tumors.

Figure 1 presents the results of ORLRS with different *θ*. Since the gene expression levels in different data are very different, the values of extreme data outliers are also very different. So, the value of *θ* has a large range in six data sets. From Figure 1, we can observe that ORLRS can obtain the best performance in the case of *θ*={10^3^, 10^5^, 40,90,400, 10^6^} in Leukemia data, DLBCL data, Colon cancer data, Brain_Tumor1 data, Brain_Tumor2 data, and 9_Tumors data, respectively. The results indicate that the value of *θ* should be determined appropriately. If the value of *θ* is too large, we will miss some extreme outliers. If the value of *θ* is too small, some important information may be removed, thereby affecting the clustering performance.

#### 3.3.2. Determination of Balance Parameter *λ*

**Figure 2:**
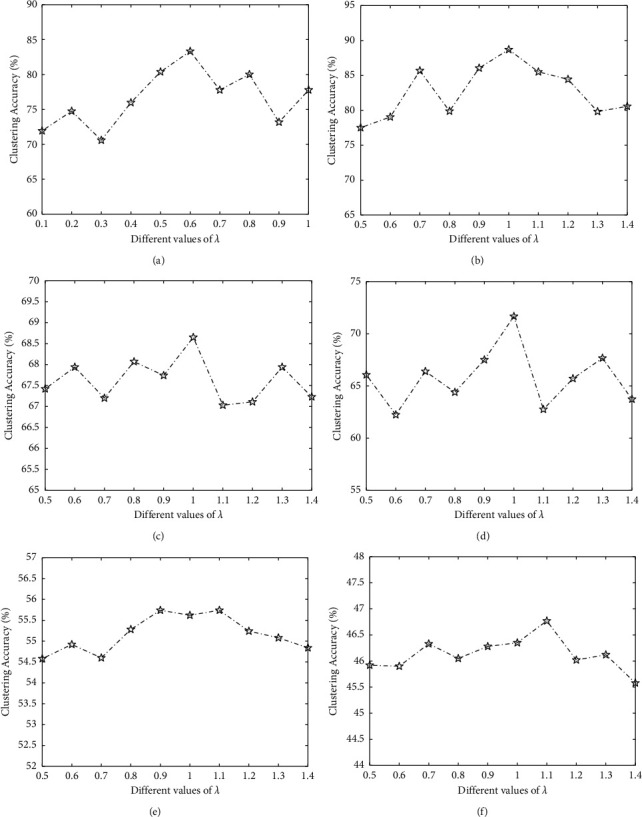
Clustering accuracies of the proposed ORLRS on six data sets with different values of *λ*. (a) Leukemia. (b) DLBCL. (c) Colon. (d) Brain_Tumor1. (e) Brain_Tumor2. (f) 9_Tumors.

Figure 2 presents the results of ORLRS with different *λ*. ORLRS can obtain the best results in the case of *λ*={0.6, 1,1,1,0.9, 1.1} in Leukemia data, DLBCL data, Colon cancer data, Brain_Tumor1 data, Brain_Tumor2 data, and 9_Tumors data, respectively. According to the experimental results in each data set, before reaching the best results, the clustering accuracies showed an overall upward trend when *λ* increases; after achieving the best results, the clustering accuracies showed an overall downward trend when *λ* increases. So, we suggest a rough range 0.1 ≤ *λ* ≤ 2 on the choice of *λ*.

#### 3.3.3. Determination of Schatten P-Norm Parameter *k*

**Figure 3:**
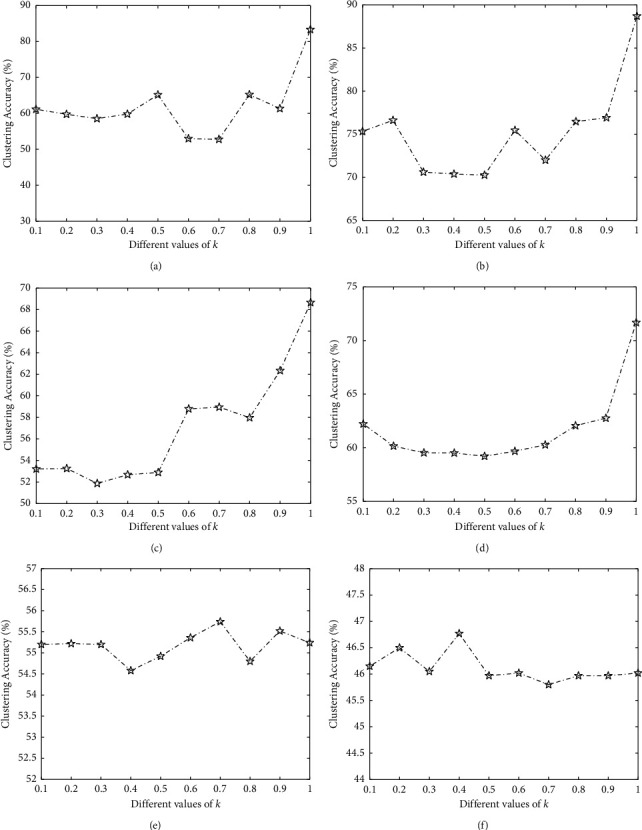
Clustering accuracies of the proposed ORLRS on six data sets with different values of *k*. (a) Leukemia. (b) DLBCL. (c) Colon. (d) Brain_Tumor1. (e) Brain_Tumor2. (f) 9_Tumors.

Since the algorithm is converged for the Schatten *p*-norm parameter 0 < *k* ≤ 1, we determine the value of *k* in this range.  Figure 3 presents the results of ORLRS with different *k*. ORLRS can achieve the best performance in the case of *k*={1,1,1,1,0.7, 0.4} in Leukemia data, DLBCL data, Colon cancer data, Brain_Tumor1 data, Brain_Tumor2 data, and 9_Tumors data, respectively. So, a general guidance is given 0 < *k* ≤ 1 on the choice of *k*.

### 3.4. Experimental Results

In this section, experimental results of our proposed method and six comparison algorithms, i.e., LRS, Ext-LRR, RPCA, PLRR, robust LRR, LatLRR, robust NMF, and K-means, are reported. ORLRS, LRS, and Ext-LRR use *K*-means to initialize the indicator matrix **I**_*i*_*|*_*i*=1_^*c*^. PLRR, robust LRR, and LatLRR use the normalized cuts method to segment data, which cluster data points by using the *K*-means method. For robust NMF method, we initial the coefficient matrix and basis matrix randomly. To avoid randomness, we run all methods 100 times, and the mean and standard error results of the clustering accuracies of 100 times are shown in Table 2. The best result of each data is indicated in bold.

Based on the results reported in Table 2, we have the following observations and discussions. ORLRS extends LRS by adding a noise matrix into the objective function to enhance the robustness, which contributes to the observation that ORLRS outperforms LRS. From the results shown in Table 2, it can be observed that ORLRS achieves generally 8%–19% higher performances than LRS in terms of the clustering accuracy on four data sets, i.e., Leukemia data, DLBCL data, Colon cancer data, and Brain_Tumor1 data. On Brain_Tumor2 and 9_Tumors data sets, ORLRS has a slightly better performance than LRS. ORLRS has better results than Ext-LRR on all datasets. Compared to the three classical low-rank based methods, PLRR, robust LRR, and LatLRR, the clustering accuracy of ORLRS is 1%–9% higher on all six data. The main reason is that we use capped norm to remove the extreme outliers in the noise matrix and Schatten *p*-norm to better approximate the low-rank representation. Compared with traditional clustering methods, RPCA, robust NMF, and K-means, ORLRS achieves outstanding results on all of the six data sets.

The NMI results on five gene expression data sets are shown in Table 3. The best result of each data is indicated in bold. Due to the NMI results of all the methods on colon data is less than 0.1, we only reported the results of remain five data sets. From Table 3, we can observe that ORLRS has better results on all the five data sets than PLRR, robust LRR, LatLRR, robust NMF, RPCA, and Ext-LRR. Except on 9_Tumors data set, our method outperforms LRS and K-means on the other four data sets.

### 3.5. Convergence Curves and Running Time

**Figure 4:**
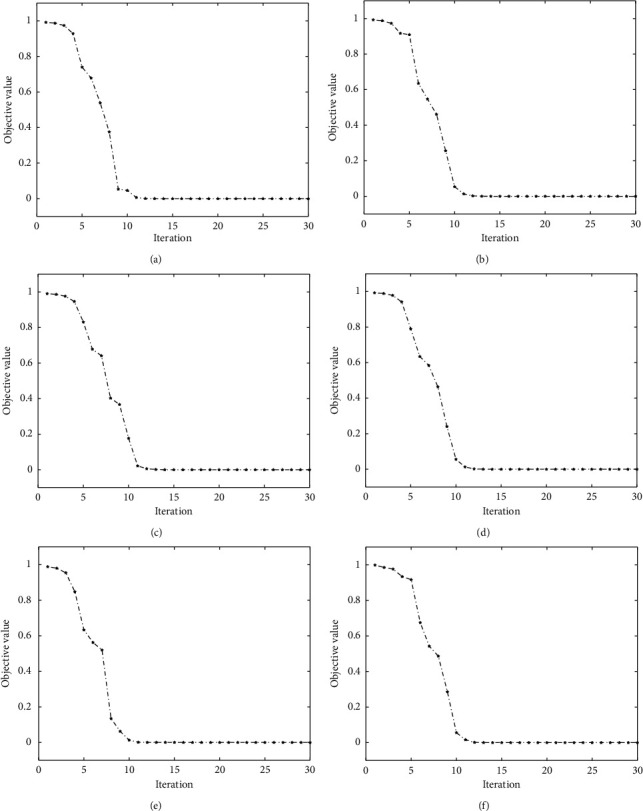
The convergence of ORLRS on six data sets. (a) Leukemia, (b) DLBCL, (c) Colon, (d) Brain_Tumor1, (e) Brain_Tumor2, (f) 9_Tumors.

We plotted the convergence curves of our ORLRS on different datasets. The convergence curves can be found in Figure 4. It shows that our method can converge around the 10-th iteration on all six data sets. In Table 4, we also reported the running time of ORLRS on six gene expression data sets without dimensionality reduction by PCA. We implement our experiment with MATLAB R2020b on an ordinary computer, which is configured with Intel i9-10900 KF (up to 3.70 GHz) cores, 8 GB RAM, and Windows 10 operating system.

## 4. Conclusions

In this paper, a novel one-step robust low-rank subspace clustering method (ORLRS) is proposed for tumor clustering, where the gene expression data set is represented by a low-rank matrix and a noise matrix. By using the Schatten *p*-norm and discrete constraint, low-rank representation of each subspace can be well obtained. Different from traditional low-rank-based methods, such as LRR and LatLRR, ORLRS learns indicators directly and perform clustering process in one step by using the discrete constraint. Capped norm is used to improve the robustness of ORLRS since it can effectively remove the extreme data outliers in the noise matrix. Furthermore, we propose an efficient algorithm to solve the proposed subspace clustering model, and the convergence of the proposed algorithm is proved. We thus can discover the clusters of tumor data depending on the optimal cluster indicators. We tested the proposed ORLRS method on six tumor data. The results are proved that ORLRS is an excellent method for clustering tumor sample.

There remain several interesting directions for future work. First, it might be better to learn a dictionary for ORLRS since some low-rank subspace segmentation methods achieve significant improvements by learning a dictionary. Second, ORLRS may be extended to solve other problems, such as matrix recovery and classification. Third, ORLRS may be employed in other applications, such as gene clustering and coclustering.

## Figures and Tables

**Algorithm 1:**
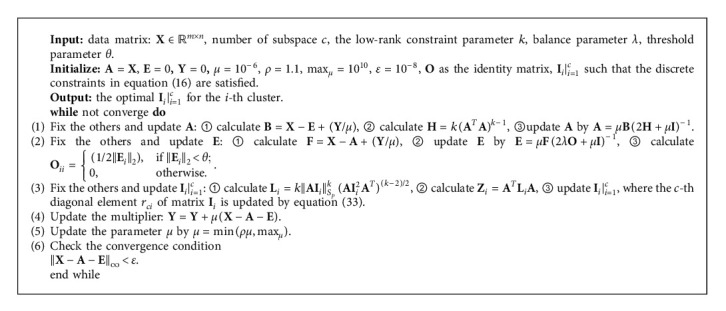
An efficient algorithm to solve the problem in equation (4).

**Table 1:** Summary of tumor gene expression data sets.

Data set	Diagnostic task	Number of		
		Genes	Samples	Classes
Leukemia	AML, ALL	7129	72	2
DLBCL	DLBCL, FL	5469	77	2
Colon cancer	Tumor, normal	2000	62	2
Brain_Tumor1	Medulloblastoma, malignant glioma, normal cerebellum, AT/RTs, PNETs	5920	90	5
Brain_Tumor2	CG, CAO, NCG, NCAO	10367	50	4
9_Tumors	NSCLC, colon, breast, ovary, renal, leukemia, melanoma, prostate, CNS	5726	60	9

**Table 2:** Clustering accuracy (%) and standard error (%) of different methods on six gene expression data sets.

Data sets	Methods								
	ORLRS	LRS	PLRR	Robust LRR	LatLRR	Robust NMF	*K*-means	RPCA	Ext-LRR
Leukemia	**83.33** ± **0.00**	74.63 ± 2.14	74.57 ± 2.56	63.89 ± 0.00	66.67 ± 0.00	66.25 ± 5.03	68.06 ± 2.93	46.69 ± 0.00	65.28 ± 0.00
DLBCL	**88.70** ± **3.50**	76.62 ± 0.00	79.35 ± 0.41	76.62 ± 0.00	76.62 ± 0.00	68.96 ± 7.71	68.83 ± 0.00	57.49 ± 0.00	73.64 ± 2.87
Colon	**74.63** ± **2.14**	55.16 ± 1.59	72.79 ± 2.87	60.81 ± 1.09	58.06 ± 0.00	70.65 ± 7.25	53.71 ± 2.12	39.71 ± 0.00	64.52 ± 0.00
Brain_Tumor1	**71.67** ± **2.30**	60.22 ± 4.53	69.22 ± 10.32	68.89 ± 0.00	69.69 ± 2.19	66.11 ± 2.58	43.78 ± 1.83	57.67 ± 0.00	67.00 ± 4.86
Brain_Tumor2	**55.74** ± **2.93**	55.36 ± 2.51	54.20 ± 2.36	44.00 ± 0.00	50.00 ± 0.00	34.00 ± 2.98	39.20 ± 1.03	46.00 ± 0.00	48.40 ± 4.60
9_Tumors	46.77 ± 4.62	45.85 ± 2.90	41.67 ± 1.05	38.67 ± 1.05	43.33 ± 0.00	35.50 ± 3.60	45.52 ± 3.16	33.33 ± 0.00	31.67 ± 2.83

**Table 3:** NMI and standard error of different methods on five gene expression data sets.

Data sets	Methods								
	ORLRS	LRS	PLRR	Robust LRR	LatLRR	Robust NMF	*K*-means	RPCA	Ext-LRR
Leukemia	0.1347 ± 0.0928	0.1025 ± 0.0466	0.1229 ± 0.0586	0.0684 ± 0.0000	0.0684 ± 0.0000	0.0920 ± 0.0529	0.1250 ± 0.0534	0.0275 ± 0.0000	0.1042 ± 0.0778
DLBCL	0.1387 ± 0.1113	0.1104 ± 0.0209	0.1208 ± 0.1020	0.0937 ± 0.0000	0.0937 ± 0.0000	0.0188 ± 0.0000	0.1150 ± 0.0141	0.0188 ± 0.0000	0.0853 ± 0.1617
Brain_Tumor1	0.3854 ± 0.0743	0.3045 ± 0.0538	0.3689 ± 0.0773	0.2914 ± 0.0146	0.2864 ± 0.0464	0.3254 ± 0.0786	0.2854 ± 0.0534	0.0362 ± 0.0000	0.2846 ± 0.0675
Brain_Tumor2	0.5486 ± 0.0431	0.5407 ± 0.0461	0.5415 ± 0.0451	0.3668 ± 0.0257	0.2645 ± 0.0323	0.2935 ± 0.0565	0.5208 ± 0.0370	0.1296 ± 0.0000	0.2913 ± 0.0867
9_Tumors	0.4082 ± 0.0463	0.4294 ± 0.0421	0.3885 ± 0.0664	0.3832 ± 0.0424	0.3854 ± 0.0497	0.2593 ± 0.0487	0.4204 ± 0.0222	0.2839 ± 0.000	0.2923 ± 0.0246

**Table 4:** The running time of ORLRS on six gene expression data sets.

Data sets	Leukemia	DLBCL	Colon	Brain_Tumor1	Brain_Tumor2	9_Tumors
Running time (second)	652.35	358.36	121.73	385.64	990.87	370.34

## Data Availability

The data used to support the findings of this study are available from the first author upon request.
